# Taxonomic inflation as a conservation trap for inbred populations

**DOI:** 10.1111/eva.13677

**Published:** 2024-05-08

**Authors:** Miguel Clavero, Javier Naves, María Lucena‐Perez, Eloy Revilla

**Affiliations:** ^1^ Estación Biológica de Doñana – CSIC Sevilla Spain

**Keywords:** Cantabrian capercaillie, extinction vortex, genetic rescue, inbreeding depression, subspecies, taxonomic inflation, *Tetrao urogallus*

## Abstract

Conservation is prioritized based on accepted taxa. As a consequence, a conservation incentive exists to emphasize inter‐population differences to define taxa, potentially leading to taxonomic inflation. But stressing the uniqueness of threatened populations has the side effect of hindering conservation actions that promote inter‐population gene flow, such as genetic rescue. These actions may be of critical importance for severely inbred populations involved in extinction vortices, for which an inflated taxonomy can become a conservation trap. Here, we exemplify this scenario with the western capercaillie (*Tetrao urogallus*, Phasianidae) population in the Cantabrian Mountains, described and legally listed as a subspecies not supported by recent molecular data. The Cantabrian capercaillie population is Critically Endangered after a long‐lasting decline and a recent demographic collapse. It shows clear signs of inbreeding depression, including striking clutch size decreases as well as reduced hatchability and chick survival. This critical situation could be alleviated through a genetic rescue, but this possibility is hindered by inertias rooted in the putative uniqueness of the Cantabrian capercaillie. It had been previously argued that poor taxonomy could hamper conservation, through the oblivion of populations deserving, but not having, a taxonomic status. We show that taxonomic inflation can also become an obstacle for effective conservation.

## 1 INTRODUCTION

The conservation of biodiversity largely relies on a taxonomic backbone, whose basic units, species and subspecies, are the core of conservation priorities and legal instruments (Garnett & Christidis, 2017). It is generally assumed that acquiring a unique taxonomic status, that is becoming a named species or subspecies, favours the conservation of threatened populations, by scoring higher in the prioritization of conservation needs. Alternatively, conservation efforts on a given set of populations may be limited if they are not recognized as a taxonomic unit (Morrison III et al., 2009). However, as we discuss here, the damaging effects of poor taxonomy may not be limited to undescribed taxa, but may arise also from overstating differences resulting in an excessive number of recognized taxa, the so‐called taxonomic inflation (Berrilli et al., 2024; Padial & De la, 2006).

Isolated populations that have declined due to extrinsic factors (e.g. overexploitation, habitat destruction or fragmentation) may suffer inbreeding depression, through which reduced genetic diversity and increased inbreeding produce negative fitness effects (Harrisson et al., 2019; Ralls et al., 2018). Inbreeding depression may generate a self‐fuelled, intrinsic process known as extinction vortex that may drive further declines and genetic deterioration and lead to the extinction of inbred populations (Fagan & Holmes, 2006). On top of this, extrinsic threats may still accelerate the decline of a population experiencing an extinction vortex.

Inbred populations may benefit from genetic rescue operations, aimed at restoring genetic diversity and overcoming deleterious effects of inbreeding depression by allowing gene flow from healthy populations (Hedrick & Garcia‐Dorado, 2016). But when an endangered population is identified as a unique taxon, the implementation of management actions involving its mixing with other populations will be hindered (Liddell et al., 2021). If the claims for uniqueness are based on an inflated taxonomy, the associated hinderances for specific conservation actions are misleading. We argue that this is may happen often when conservation planning uses a subspecies‐level taxonomic template.

Subspecies descriptions were profusely produced in the late‐19th century and across the 20th century but are notably less frequent in recent decades (Zink & Klicka, 2022). The description of subspecies has had large biases, both geographically (being particularly frequent in Europe and North America) and taxonomically (covering mainly mammals, birds and butterflies) (Burbrink et al., 2022). On top of these biases, subspecies descriptions often had poor bases and are not currently supported (e.g. Dufresnes et al., 2023), particularly in continental regions of Europe and North America (Phillimore & Owens, 2006). For example, the hundreds of subspecies that had been proposed for the brown bear (*Ursus arctos*) were progressively reduced to 9, which still do not match the seven genetic lineages described within the species (Hall, 1984; Kitchener et al., 2020).

Lists of protected taxa often include unsupported subspecies as an inheritance of past taxonomic inflation inertias. This introduces a burden for biodiversity conservation (Zink & Klicka, 2022) and may hinder specific conservation actions, including genetic rescue operations. For example, the unique taxonomic status of the Florida panther (putatively, *Puma concolor coryi*) was one of the main arguments against genetic rescue of that severely inbred population (Pimm et al., 2006), even though the 15 North American puma subspecies that had been described actually belong to a single genetic lineage (Culver et al., 2000). Here, we develop the idea that taxonomic inflation can act as a conservation trap, using the western capercaillie (*Tetrao urogallus*) as an example.

## 2 CAPERCAILLIE INTRASPECIFIC VARIATION

**FIGURE 1:**
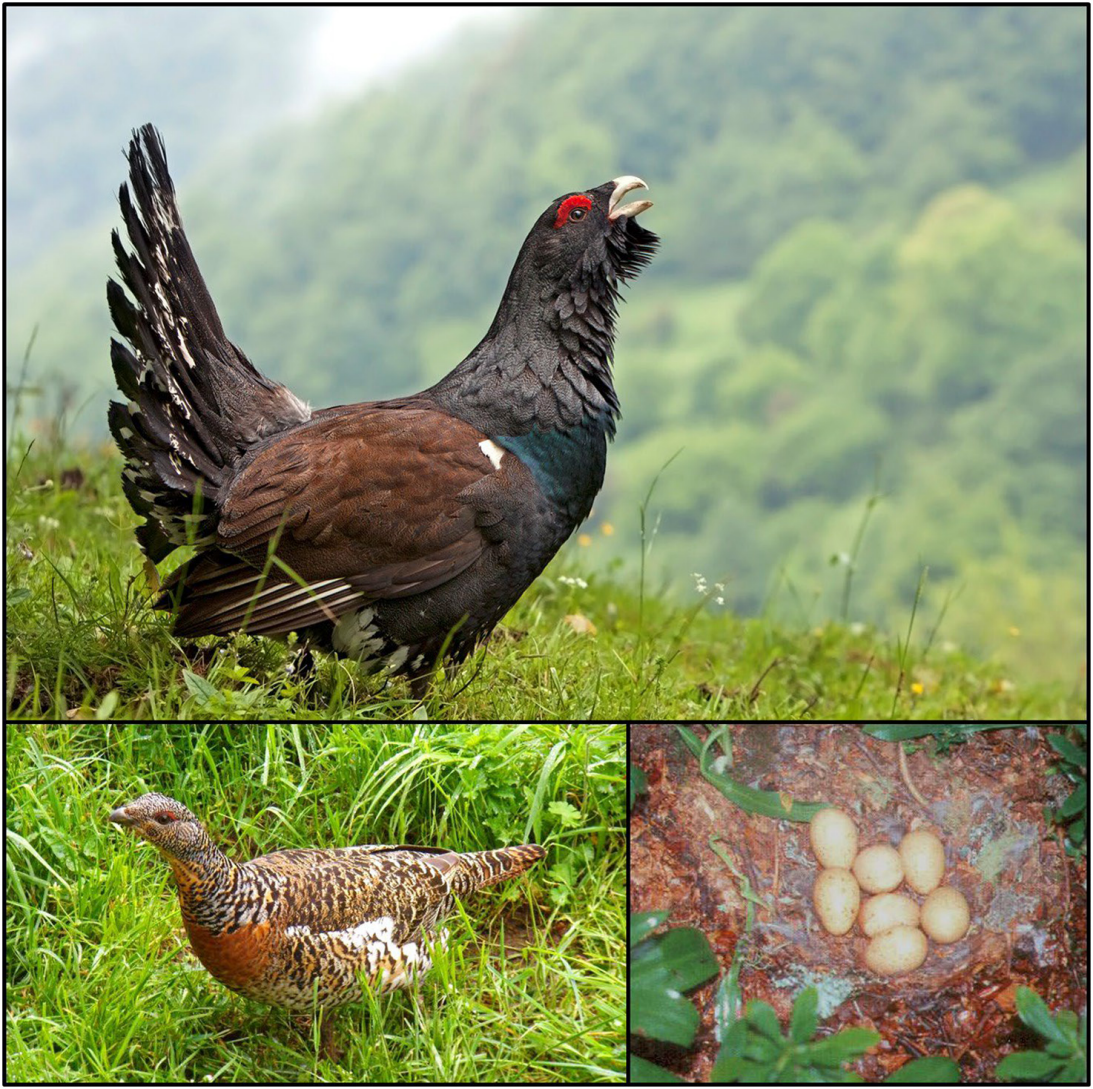
The Cantabrian capercaillie. Above, a male individual (photo: J.L. Rodríguez, hosted at the gallery of Urogallo Cantabrico Life+ project). Bottom left, a female (photo: L. Robles, hosted at the gallery of Urogallo Cantabrico Life+ project). Bottom right, a capercaillie nest with seven eggs, photographed in 1996 by C. Pollo and included in Figure 2.

The western capercaillie (Figure 1; henceforth, simply capercaillie) is the largest extant grouse species (de Juana, 1994). It is a forest specialist that forages and nests on the ground. It generally inhabits coniferous forests with an Ericaceae (*Vaccinium*, *Calluna*) understory. However, some populations (e.g. in Iberian Peninsula or the Urals) can occupy deciduous forests (de Juana, 1994). The species has a marked sexual dimorphism, males being notably larger and more contrastingly coloured than females (Figure 1). The courtship display of the capercaillie involves male leks visited by females, which thereafter take full care of nests and chicks.

**FIGURE 2:**
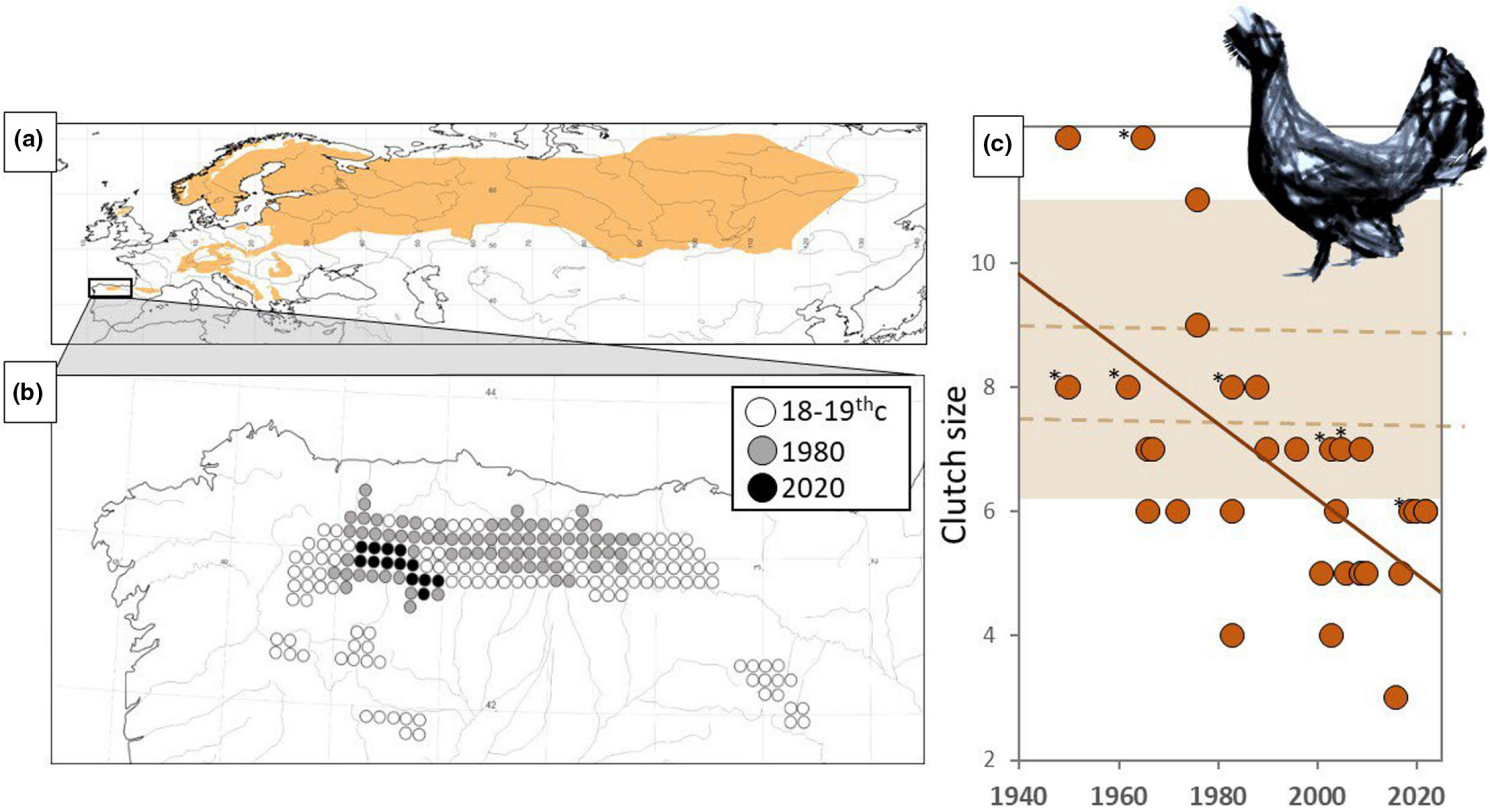
(a) Present range of the western capercaillie (*Tetrao urogallus*), as reported by the IUCN Red List. The Cantabrian range is framed with a rectangle and enlarged below. (b) Range reduction of the Cantabrian capercaillie, showing the estimated historical range (18–19th centuries), the 1970s range (these two from Rodríguez‐Muñoz, 2011) and the situation in 2020 (Jiménez et al., 2022). Circles correspond to UTM 10 km cells. (c) Temporal evolution of clutch size of the Cantabrian capercaillie, from data on 32 nests with eggs or young chicks recorded in the field (data in Table S1). * Denotes that the data refer to groups of unfeathered chicks (i.e. less than 10 days of age). The regression line equation (*y* = 127.1–0.0605×) involves that the average clutches have lost one egg every 16.5 years since 1950 (*R* ^2^ = 0.40; *p* < 0.001). The shaded background area shows the range of mean clutch size values reported in the literature, while dotted lines represent central values of the ranges reported in monographies (data in Table S2).

The wide distribution of the capercaillie, covering from Spain to Eastern Russia, is fragmented into multiple isolated and threatened populations at its western limits (Figure 2a, BirdLife International, 2021). Across this range, up to 13 subspecies have been described, mainly based on morphological and coloration features (de Juana, 1994). The Cantabrian subspecies (*T. urogallus cantabricus*) was described in the 1960s, based on a smaller beak and a darker coloration than the putative Pyrenean subspecies (*T. urogallus aquitanus*), with the individuals of both populations being smaller than those of other subspecies (Castroviejo, 1967).

**FIGURE 3:**
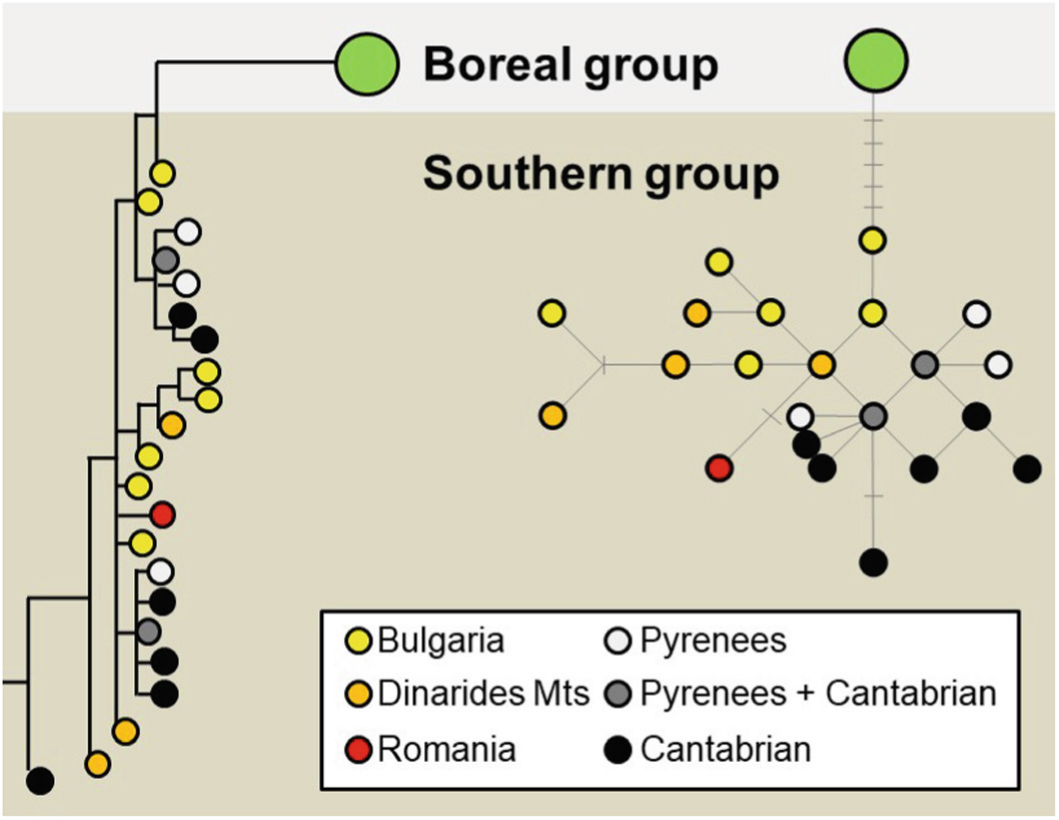
Genetic patterns in the wester capercaillie, summarized and adapted from Figure 1 in Bajc et al. (2011) to highlight diversity within the southern group (or lineage). Results are based in the sequencing the first 435 nucleotides of the mitochondrial DNA Control Region. The left graph shows a maximum likelihood tree and the right one a minimum spanning haplotype network. Graphs show that the Cantabrian capercaillie is not an independent lineage.

The infraspecific taxonomy of the western capercaillie has not been supported by molecular data (Figure 3). Based on the variability of the mtDNA Control Region, two groups are generally described, namely boreal and southern, the latter being the most common in mountain ranges of southern Europe (Duriez et al., 2007). However, the two groups are not monophyletic, because the boreal group is nested within the southern one (Figure 3, Bajc et al., 2011). The limited and sparse available information on nuclear DNA suggests that the current spatial patterns of mtDNA variability do not reflect independent evolutionary lineages and could have resulted from a recent and generalized decline of the species across its European range (Rodríguez‐Muñoz et al., 2007).

## 3 CAPERCAILLIE DECLINE AND INBREEDING DEPRESSION

The present range of the Cantabrian capercaillie, around 1000 km^2^, is only 15% of what it was in the early 1970s and less than 5% of the estimated 19th century range (Figure 2b). On top of the spatial decline, the number of individuals has also shrunk dramatically, with an estimated loss of 90% between 1978 and 2019 (Jiménez et al., 2022). Intense legal and illegal hunting pressure has predominately targeted males and has likely been the main driver in the decline of the Cantabrian capercaillie (Rodríguez‐Muñoz et al., 2015; Vigil, 2014). Other factors, such as habitat modifications, could have been involved in this decline, but it is worth noting that the collapse of the Cantabrian capercaillie has occurred in parallel with a generalized increase in forest cover across its historical range (e.g. Pisabarro et al., 2019).

The decline of Cantabrian capercaillie is concomitant to a low genetic diversity and high inbreeding in the population (Escoda et al., 2023; Rodríguez‐Muñoz et al., 2007). Furthermore, data on 32 clutches located in the field reveal that average clutch size has declined by almost 50% in the last 70 years (Figure 2c, data and details in Table S1). On top of the reduction in egg production, hatchability and chick survival seems to be severely impaired. For example, the ex situ Cantabrian capercaillie breeding programme informed that out of 204 eggs have been laid between 2010 and 2017, only 56 hatched and 23 chicks survived (Life Urogallo Cantábrico, 2016). O'Brien et al. (2023) found that up to one third of semen samples from a mixed pool of Cantabrian and other European capercaillies were azoospermatic, a feature that was not observed in chicken (*Gallus gallus domesticus*) or red‐legged partridges (*Alectoris rufa*) within the same sampling protocol. The same authors also reported notably lower spermatozoid densities (around 20 times smaller) in capercaillie than in the other studied species.

Inbreeding depression hindering successful reproduction may arguably be a cause of total lack of positive effects of every ex situ and in situ management action implemented to date. The former have mainly involved habitat management (basically understorey clearings) and predator control (MMA, 2004), while the later has led to the construction of two captive breeding facilities (in 2009 and 2022). Many of these activities have been supported by a Life+ project, but its executive summary states that ‘there is no proof that the decline in the species has been halted and there seems to be a downward trend’ (Life + Urogallo Cantábrico, 2016; see also Robles et al., 2021). This lack of management results and the worsening of the status of the Cantabrian capercaillie led to its Critically Endangered categorization in the Spanish legislation (BOE, 2018), although this has not involved any relevant change in the main management strategies (e.g. MITECO, 2020). The long‐term population decline and poor management success are in fact common features of several isolated capercaillie populations (e.g. in the Pyrenees; Gil et al., 2020).

Surprisingly, the possible influence of poor genetic conditions on the decline of European capercaillie populations has been sparsely (at best) mentioned in the literature (e.g. BirdLife International, 2021, but see Quevedo et al., 2005). Jahren et al. (2016) reviewed the declining reproductive output of capercaillie across Europe, but did not discuss possible inbreeding depression, likely because of the lack of appropriate field data, which are in general scarce for wild populations (Höglund et al., 2002; but see Briskie & Mackintosh, 2004, Harrisson et al., 2019). Typically, capercaillie populations are characterized through reproduction‐related parameters recorded through field observations of juveniles, such as nest success and broods or chicks per hen (see Jahren et al., 2016). Those parameters are the result of a series of concatenated processes, including clutch size, nest predation, hatching success and chick survival, which cannot be properly characterized through the observation of juvenile birds. In particular, there is a dearth of field data series on clutch size, especially after the 1990s (Table S2). Notable exceptions are data from Slovakia (Saniga, 1996, 2011) and the reintroduced population in Scotland (Proctor & Summers, 2002). In both cases, data show temporal declines in clutch size.

## 4 MANAGING INBREEDING DEPRESSION

The effects of inbreeding depression have been known for a long time and are well documented in Galliformes, especially within the poultry industry (Stephenson et al., 1953). Captive‐bred populations of wild Galliformes are also impaired by inbreeding depression, affecting clutch size, hatchability and chick survival (Hammerly et al., 2016). Genetic diversity and inbreeding have been assessed in some wild Galliform populations (e.g. Davis et al., 2015; Kirchman et al., 2020). A particularly well‐known case involved the loss of genetic diversity due to the decline of the Illinois greater prairie‐chicken (*Tympanuchus cupido pinnatus*), which was followed by a steep decline in egg fertility and hatching success (Westemeier et al., 1998). Genetic rescue involving translocations of individuals from large, genetically diverse populations produced immediate improvements of fitness parameters of this threatened taxon, reverting population declines (Westemeier et al., 1998) and allowing the recovery of genetic diversity (Capel et al., 2022).

Genetic rescue is usually seen as an urgent and extreme measure, as it could result in a fitness reduction due to an influx of maladaptive alleles or the disruption of co‐adapted gene complexes (i.e. outbreeding depression, Frankham et al., 2011). However, accumulated evidence shows that genetic rescue has more positive than negative effects and that the reduction of inbreeding depression outweighs possible outbreeding risks (Fitzpatrick et al., 2020). Hence, genetic rescue should be considered in those cases in which species persistence is severely compromised by inbreeding depression and when a suitable source population exists, even considering the possibility of admixing distinct subspecies (Harrisson et al., 2016).

The Cantabrian capercaillie requires a genetic rescue initiative to alleviate inbreeding depression, increase fitness and revert demographic trends. Even if habitat management and effective protection will be needed in the long‐term (assuming that extinction is avoided), there is an urgent need to restore the fitness parameters of the Cantabrian capercaillie by alleviating inbreeding depression. However, managing wild populations in general, and grouse ones in particular, is challenging and genetic rescue should not be seen as a silver bullet. Despite uncertainties, genetic rescue is an unavoidable management step in the conservation of the Cantabrian capercaillie. However, after the decades‐long stressing of the uniqueness of the Cantabrian capercaillie as a core reason to prioritize its conservation, its admixture with birds from different populations has been clearly hindered (Martínez‐Abraín et al., 2022). In this sense, the acceptance of the inflated intraspecific taxonomy of the capercaillie as a conservation argument has become a conservation trap.

## 5 CONCLUDING REMARKS

A cross‐cutting aim of environmental management should be conserving the largest possible amount of biological diversity. This general target involves maintaining the integrity of evolutionary units with their unique genetic, behavioural and ecological adaptations to local or regional environments (Hoban et al., 2021). These aspects have been often overlooked by management actions involving stockings, leading to genetic homogenization and losses of local lineages through hybridization (e.g. Casas et al., 2012). However, not all genetic groups described through the analysis of contemporary genetic patterns have an intrinsic conservation value. For example, apparent genetic divergence may not result from long‐lasting evolutionary processes involving adaptive evolution, but from recent species declines, most often related to anthropogenic impacts (Lucena‐Perez et al., 2020). These situations are not uncommon and, if ignored, could involve sterile complexities for the conservation of biodiversity, especially when the recent genetic clusters have been described as unique taxa.

Considering the limited success of capercaillie captive breeding so far (Storch, 2007) and the critical status of the Cantabrian population, genetic rescue should favour the translocation of individuals (see Storch, 2007 and references cited therein). The opportunities and shortcomings of species translocations, including though not limited to genetic rescue, have been widely debated in the literature (e.g. Bell et al., 2019; Brodie et al., 2021). Generally, we agree with the precautionary advice often associated with this kind of aggressive interventions, which can have unintended deleterious impacts. However, when precaution is founded over misleading taxonomic inflation, as in the case of the Cantabrian capercaillie, it may promote inaction and ultimately favour the extinction of populations with overstated uniqueness. Conserving biodiversity is a complex issue and bad taxonomy can actually kill, by both fault and excess.

## CONFLICT OF INTEREST STATEMENT

The authors declare that they do not have any conflict of interest related to the content of this publication.

## Supporting information


Table S1.
Table S2.

## Data Availability

All data are provided as supplementary materials.
