# Supplementary material for: Taxonomic inflation as a conservation trap for inbred populations
Source: Evol Appl. 2024 May 8;17(5):e13677. doi: 10.1111/eva.13677 (PMC11078296; doi:10.1111/eva.13677)
Supplement: Supplementary file 1 — Table S1. Table S2. [file EVA-17-e13677-s001.docx]

**Taxonomic inflation as a conservation trap: the capercaillie case**

**SUPPLEMENTARY MATERIALS**

TABLE S1. Number of eggs or young chicks (*) found in 32 Cantabrian capercaillie nests between 1950 and 2022.

| **year** | **clutch size** | **Area (province)** | **Source** |
| --- | --- | --- | --- |
| 1950 | 8 * | Los Tojos (Cantabria) | hunter (reported by AV) |
| ~ 1950 | 12 | Vega de Liebana (Cantabria) | hunter (reported by AV) |
| ~ 1962 | 8 * | Piloña (Asturias) | wildlife ranger (reported by AV) |
| 1965 | 11 * | Caso (Asturias) | wildlife ranger (reported by AV) |
| 1966 | 6 | Ancares area (Lugo) | Castroviejo (1975) |
| 1966 | 7 | Ancares area (Lugo) | Castroviejo (1975) |
| 1967 | 7 | Ancares area (Lugo) | Castroviejo (1975) |
| 1972 | 6 | Palacios del Sil (León) | hunter (reported by HR) |
| ~ 1976 | 9 | RNC Fuentes Carrionas (Palencia) | Ortuño & de la Peña (1977) |
| ~ 1976 | 11 | RNC Fuentes Carrionas (Palencia) | Ortuño & de la Peña (1977) |
| 1983 | 6 | Posada de Valdeón (León) | wildlife technician (reported by MG) |
| 1983 | 4 | Posada de Valdeón (León) | wildlife technician (reported by MG) |
| ~ 1983 | 8 * | Villafranca del Bierzo (León) | hunter (reported by AV) |
| 1988 | 8 | Tresviso (Cantabria) | Fernández-Gil et al (1989) |
| 1990 | 7 | Somiedo (Asturias) | Naves et al. (1990) |
| 1996 | 7 | Posada de Valdeón (León) | wildlife technician (reported by JN) |
| 2001 | 5 | Palacios del Sil (León) | wildlife technician (reported by JN) |
| 2003 | 4 | Allande (Asturias) | González-Quirós et al. (2003) (reported by JN) |
| 2003 | 7 * | Villablino (León) | wildlife ranger (reported by JN) |
| 2004 | 6 | Vega de Liebana (Cantabria) | wildlife technician (reported by JN) |
| 2006 | 5 | Cangas del Narcea (Asturias) | Rodríguez-Muñoz (2011) |
| ~ 2005 | 7 * | Cangas del Narcea (Asturias) | wildlife technician (reported by JN) |
| 2009 | 7 | Omañas area (León) | González (2012) |
| 2009 | 5 | Quintana del Castillo (León) | González (2012) |
| 2009 | 5 | Cangas del Narcea (Asturias) | Granda (2009) |
| 2010 | 5 | Villablino (León) | GTUC (2013) (reported by JN) |
| 2016 | 3 | Villablino (León) | LIFE+ 09 NAT/ES/513 (2016) (reported by JN) |
| 2017 | 5 | Cangas del Narcea (Asturias) | wildlife technician (reported by JN) |
| 2019 | 6 * | Ancares area (León) | GTUC (2019) |
| 2020 | 6 | Ancares area (León) | GTUC (2020) |
| 2022 | 6 | Villablino (León) | CMA (2022) (reported by JN) |
| 2022 | 6 | Villablino (León) | CMA (2022) (reported by JN) |

*unfeathered chicks, also noted in Figure 1C

Sources:

AV- Adrián Vigil

MG- Manuel Antonio González

JN- Javier Naves

HR- Héctor Ruiz-Villar

Castroviejo J (1975) *El urogallo en España*. Monografías de la Estación Biológica de Doñana, 3. Consejo Superior de Investigaciones Científicas, Madrid.

CMA (2022). Press release: La Junta convierte, con una inversión de más de 2 millones de euros, el nuevo centro de cría del urogallo de Valsemana, en León, en referencia internacional en el ámbito de la investigación. Junta de Castilla y León.

Fernández_Gil A et al (1989) *Dinámica de las poblaciones de urogallo Cantábrico en la Reserva Nacional de Caza de Saja*. Report of Departamento de Geografía, Urganismo y Ordenación del Territorio. Universidad de Cantabria, Santander.

González MA (2012). *Biology and Conservation of the Capercaillie in a Mediterranean environment*. PhD thesis. Universidad de Léon, Spain.

González-Quirós et al (2003). *Seguimiento de una hembra de urogallo radiomarcada en Allande (Asturias) (Mayo-agosto de 2003)*. Report to Consejería de Medio Ambiente. Principado de Asturias

Granda C (2009) *Informe actuaciones*. Report to Direccion General de Biodiversidad y Paisaje. Consejería Medio Ambiente y Desarrollo Rural. Principado de Asturias

Grupo de trabajo del Urogallo Cantábrico (2013). Protocolo de actuación para la captura y radiomarcaje de hembras de urogallo. Ministerio para la Transición Ecológica y el Reto Demográfico. Gobierno de España

GTUC (Cantabrian capercaillie working group) (2019) *Acciones urgentes in situ para la recuperación de especies declaradas en situación crítica: Urogallo Cantábrico. Informe de resultados. Anualidad 2019.* Report to Ministerio para la Transición Ecológica y el Reto Demográfico. Gobierno de España

GTUC (Cantabrian capercaillie working group) (2020). *Acciones urgentes in situ para la recuperación de especies declaradas en situación crítica: Urogallo Cantábrico. Informe de resultados. Anualidad 2020*. Report to Ministerio para la Transición Ecológica y el Reto Demográfico. Gobierno de España

Naves et al (1990). Censo de urogallo (*Tetrao urogallus cantabricus*) en el Parque Natural de Somiedo. Gabinete de Estudios Ambientales. Principado de Asturias

Ortuño F & de la Peña J (1977) *Reservas y Cotos Nacionales de Caza. 2 Región Cantábrica*. Incafo, Madrid

Proyecto LIFE+ 09 NAT/ES/513 (2016) Noticias. Nacen nueve urogallos cantábricos en cautividad. https://www.lifeurogallo.es/es/avances-del-proyecto/nacen-nueve-urogallos-cantabricos-en-cautividad

Rodríguez-Muñoz R (2011) Urogallo común – *Tetrao urogallus*. In Enciclopedia Virtual de los Vertebrados Españoles. Salvador, A., Morales, M. B. (Eds.). Museo Nacional de Ciencias Naturales, Madrid.

TABLE S2. Mean clutch size of the western capercaillie, as reported in two monographies and in works reporting field data. Whenever works reported average clutch size, we collected that data. If only ranges were reported, we took the central point of the range (i.e. the mean among the range extremes), favouring, when possible, the use of the mean range over minimum-maximum ranges. The reference year was also calculated as the central year of the study period. When this period was not known, we considered that the data had been compiled 5 years before the publication of the work.

|  | year | Clutch size | mean range | min-max | Nests | Location | Source | Period |
| --- | --- | --- | --- | --- | --- | --- | --- | --- |
| Monographies | NA | 7.5 | 6-9 | 4-12 |  |  | De Juana (1994) |  |
|  | NA | 9 | 7-11 | 5-16 |  |  | Cramp & Simmons (1980) |  |
| Rusia |  |  |  |  |  |  |  |  |
|  | 1882 | 8 | 6-8 | 12 max. |  | Central Ural | Ushkov 1887 (in Dement’ev & Gladkov 1952) |  |
|  | <1900 | 8 | 6-8 | 9-10 max. |  | Rusia and Boreal Caucasus | Ménsbir 1902 (in Sévertzov 1941) |  |
|  | 1947 | 7.6 |  | 5-16 | 22 | Southern Urals | Dement’ev & Gladkov (1952) |  |
|  | 1947 | 6.2 |  | 4-9 | 27 | Pechora | Dement’ev & Gladkov (1952) |  |
|  | 1980 | 6.35 |  |  | 146 | Pechora | Beshkarev et al. (1993) | 1976-1985 |
| Fennoscandia countries |  |  |  |  |  |  |  |  |
|  | 1947 | 6.6 |  | 4-8 |  | Lapland | Dement’ev & Gladkov (1952) |  |
|  | 1962 | 7. 2 |  |  |  | Norway | Wegge 1979 (in Proctor & Summers (2002)) | 1953-1972 |
|  | 1964 | 7.0 |  |  |  | Central Finland | Rajala 1974 (in Beshkarev et al. (1993)) | 1963-1966 |
|  | 1964 | 7.05 | 6.2-7.9 |  |  | Finland | Lindén 1981 (in Lindström (1994)) | 1952-1977 |
|  | 1965 | 7.18 | 6.3-7.8 |  | 399 | southern Norway | Selås (2000) | 1953-1978 |
|  | 1972 | 7.1 |  |  |  | Norway | Wegge 1979 (in Proctor & Summers (2002)) | 1968-1977 |
| Central and South-Eastern Europe |  |  |  |  |  |  |  |  |
|  | 1835 | 11.0 |  | 6-16 |  | Galicia and Bukovina | Zavadskii 1840 (in Dement’ev & Gladkov 1952) |  |
|  | 1848 | 11.0 |  | 6-16 |  | Carpathians | Vodzitskii 1853 (in Dement’ev & Gladkov 1952) |  |
|  | 1868 | 9.5 | 5-12 |  |  | Germany | Al'tum 1873 (in Dement’ev & Gladkov 1952) |  |
|  | 1880 | 10.0 | 8-12 |  |  | Austria | Wurm 1885 (in Dement’ev & Gladkov 1952) |  |
|  | 1965 | 7.5 |  | 6-12 | 37 | Croatia | Car (1970) (in Cramp & Simmons (1980)) |  |
|  | <1970 | 9 |  | 7-14 |  | Vosgos | Kempf et al. (1974) |  |
|  | 1973 | 7.6 |  |  | 14 | Thuringia | Klaus (1994) | 1971-1975 |
|  | 1980 | 7.5 |  |  | 13 | Thuringia | Klaus (1994) | 1976-1985 |
|  | 1987 | 8.5 |  | 6-11 | 18 | Central Slovakia | Saniga (1996) | 1981-1994 |
|  | 1990 | 6.4 |  |  | 12 | Bavarian Alps | Storch (1994) | 1988-1992 |
|  | 1995 | 6.8 |  |  | 94 | Central Slovakia | Saniga (2011) | 1981-2010 |
| Scotland |  |  |  |  |  |  |  |  |
|  | 1974 | 7.3 |  | 4-11 | 41 | Scotland | Proctor & Summers (2002) | 1950-1998 |
|  | 2005 | 7.25 |  | 6-10 | 20 | Scotland | Summers et al. (2009) | 2003-2007 |
| Iberian Peninsula |  |  |  |  |  |  |  |  |
|  | 1967 | 6.3 |  | 6-7 | 3 | Pyrenees | Castroviejo (1975) | 1966-1969 |
|  | 1970 | 7 | 6-8 | 5-10 |  | Cantabrian Mountains | Noval (1975) |  |

Sources

Beshkarev, A., Blagovidov, A., & Sokolski, S. Hjeljord, O. (1993). Populations of Capercaillie and hazel grouse in large natural and logged forests in Northern Russia, 1950-92. In 6. International grouse symposium, Udine (Italy), 20-24 Sep 1993. Istituto Nazionale per la Fauna Selvatica.

Castroviejo, J. (1975). El urogallo en España. Monografías de la Estación Biológica de Doñana, 3. Consejo Superior de Investigaciones Científicas, Madrid. 546 pp

Cramp, S., Simmons, K. E. L. (1980). Handbook of the Birds of Europe the Middle East and North Africa. The Birds of the Western Paleartic. Volume II. Hawks to Bustards. Oxford University Press, Oxford.

De Juana, E. (1994) Tetraonidae. Pp. 376-411. En: del Hoyo, J., Elliott, A., Sargatal, J. (Eds.). Handbook of the birds of the world. Vol. 2. New world vultures to guineafowl. Lynx Edicions, Barcelona.

G.P. Dement'ev, N.A. Gladkov (1952). Birds of the Soviet Union, Vol. 6, Israel Program for Scientific Translations, Jerusalem (1967), p. 683.

Klaus, S. (1994). To survive or to become extinct: small populations of Tetraonids in Central Europe. Minimum animal populations, 137-152.

Kempf Ch, Lefranc N, Pfeffer JJ, Villaume F (1974). Le Grand Tétras (*Tetrao urogallus*) dans les Vosges. Alauda 42: 17-38.

Lindström, J. (1994). Tetraonid population studies—state of the art. Pages 347–364 Annales Zoologici Fennici.

Noval A. (1975). EL libro de la Fauna Ibérica. Aves, Vol I-VI. Ediciones Naranco SA, Oviedo.

Proctor, R., Summers, R. W. (2002). Nesting habitat, clutch size and nest failure of Capercaillie Tetrao urogallus in Scotland. Bird Study, 49: 190-192.

Saniga, M. (1996). Distribution, habitat preferences and breeding biology of the capercaillie (Tetrao urogallus) population in the Velka´ Fatra mountains (West Carpathians). Biol Bratisl 51:201–211.

Saniga, M. (2011). Why the capercaillie population (Tetrao urogallus L.) in mountain forests in the Central Slovakia decline?. Folia Oecologica, 38(1), 110.

Selås, V. (2000). Population dynamics of capercaillie Tetrao urogallus in relation to bilberry Vaccinium myrtillus production in southern Norway. Wildlife Biology 6:1–11.

Sévertzov SA (1947). Dinámica de la población animal. 539 pp. Editorial Lautaro. Buenos Aires. [Original edition of the USSR Academy of Sciences. Moscow-Leningrad 1941]

Storch, I. 1994. Habitat and survival of capercaillie Tetrao urogallus nests and broods in the Bavarian Alps. Biological Conservation 70:237–243.

Summers, R. W., J. Willi, and J. Selvidge. 2009. Capercaillie Tetrao urogallus nest loss and attendance at Abernethy Forest, Scotland. Wildlife Biology 15:319–327.
